# Supplementary material for: The Importance of Anti-correlations in Graph Theory Based Classification of Autism Spectrum Disorder
Source: Front Neurosci. 2020 Aug 7;14:676. doi: 10.3389/fnins.2020.00676 (PMC7426475; doi:10.3389/fnins.2020.00676)
Supplement: Supplementary file 2 [file Table_2.DOCX]

Supplementary Material

# Craddock 200 Atlas, PCA + Graph features:

Table 1-A. Results using the unchanged correlation matrix. ACC: ACCURACY, SEN: SENSITIVITY, SPE: SPECIFICITY, AUC: Area under curve score, THRESH: Graph density threshold for the reported metrics

| **SITE** | **ACC** | **SEN** | **SPE** | **AUC** |
| --- | --- | --- | --- | --- |
| **CALTECH** | 0.613 (0.033) | 0.697 (0.049) | 0.534 (0.044) | 0.615 (0.034) |
| **CMU** | 0.681 (0.025) | 0.920 (0.035) | 0.459 (0.063) | 0.690 (0.024) |
| **KKI** | 0.544 (0.033) | 0.354 (0.066) | 0.810 (0.071) | 0.582 (0.032) |
| **LEUVEN** | 0.647 (0.035) | 0.884 (0.024) | 0.369 (0.056) | 0.626 (0.036) |
| **MAX_MUN** | 0.528 (0.021) | 0.598 (0.028) | 0.446 (0.052) | 0.522 (0.022) |
| **NYU** | 0.694 (0.014) | 0.701 (0.027) | 0.684 (0.025) | 0.693 (0.013) |
| **OHSU** | 0.601 (0.028) | 0.667 (0.073) | 0.525 (0.069) | 0.596 (0.027) |
| **OLIN** | 0.643 (0.035) | 0.577 (0.052) | 0.695 (0.048) | 0.636 (0.035) |
| **PITT** | 0.662 (0.014) | 0.793 (0.035) | 0.541 (0.024) | 0.667 (0.015) |
| **SBL** | 0.538 (0.032) | 0.792 (0.047) | 0.284 (0.036) | 0.538 (0.032) |
| **SDSU** | 0.698 (0.025) | 0.745 (0.035) | 0.623 (0.035) | 0.684 (0.025) |
| **STANFORD** | 0.516 (0.025) | 0.246 (0.043) | 0.800 (0.027) | 0.523 (0.024) |
| **TRINITY** | 0.492 (0.04) | 0.467 (0.036) | 0.520 (0.063) | 0.493 (0.041) |
| **UCLA** | 0.683 (0.017) | 0.724 (0.032) | 0.650 (0.040) | 0.687 (0.016) |
| **UM** | 0.677 (0.016) | 0.593 (0.031) | 0.771 (0.031) | 0.682 (0.016) |
| **USM** | 0.689 (0.029) | 0.775 (0.032) | 0.642 (0.043) | 0.708 (0.025) |
| **YALE** | 0.705 (0.022) | 0.723 (0.054) | 0.686 (0.034) | 0.705 (0.022) |
| **Mean** | 0.624 | 0.662 | 0.591 | 0.626 |
| **Weighted Mean** | 0.643 | 0.667 | 0.625 | 0.646 |

Table 1-B. Results using the absolute value of the correlation matrix

| **SITE** | **ACC** | **SEN** | **SPE** | **AUC** |
| --- | --- | --- | --- | --- |
| **CALTECH** | 0.58 (0.05) | 0.679 (0.053) | 0.487 (0.082) | 0.583 (0.049) |
| **CMU** | 0.626 (0.027) | 0.830 (0.061) | 0.437 (0.047) | 0.633 (0.027) |
| **KKI** | 0.572 (0.025) | 0.440 (0.035) | 0.756 (0.037) | 0.598 (0.025) |
| **LEUVEN** | 0.634 (0.014) | 0.836 (0.026) | 0.397 (0.020) | 0.617 (0.014) |
| **MAX_MUN** | 0.548 (0.017) | 0.592 (0.038) | 0.496 (0.040) | 0.544 (0.017) |
| **NYU** | 0.684 (0.016) | 0.704 (0.023) | 0.659 (0.022) | 0.681 (0.016) |
| **OHSU** | 0.523 (0.035) | 0.562 (0.033) | 0.477 (0.063) | 0.520 (0.037) |
| **OLIN** | 0.643 (0.024) | 0.548 (0.023) | 0.717 (0.031) | 0.633 (0.024) |
| **PITT** | 0.660 (0.02) | 0.743 (0.033) | 0.584 (0.031) | 0.663 (0.020) |
| **SBL** | 0.537 (0.029) | 0.807 (0.035) | 0.267 (0.057) | 0.537 (0.029) |
| **SDSU** | 0.656 (0.019) | 0.705 (0.019) | 0.579 (0.040) | 0.642 (0.021) |
| **STANFORD** | 0.533 (0.035) | 0.285 (0.060) | 0.794 (0.059) | 0.540 (0.035) |
| **TRINITY** | 0.570 (0.032) | 0.553 (0.033) | 0.590 (0.060) | 0.572 (0.033) |
| **UCLA** | 0.684 (0.021) | 0.693 (0.021) | 0.677 (0.031) | 0.685 (0.020) |
| **UM** | 0.690 (0.009) | 0.606 (0.021) | 0.784 (0.028) | 0.695 (0.010) |
| **USM** | 0.714 (0.024) | 0.764 (0.033) | 0.686 (0.039) | 0.725 (0.020) |
| **YALE** | 0.684 (0.022) | 0.808 (0.029) | 0.560 (0.041) | 0.684 (0.022) |
| **Mean** | 0.620 | 0.656 | 0.585 | 0.621 |
| **Weighted Mean** | 0.644 | 0.666 | 0.624 | 0.645 |

Table 1-C. Results using the negative correlation matrix

| **SITE** | **ACC** | **SEN** | **SPE** | **AUC** |
| --- | --- | --- | --- | --- |
| **CALTECH** | 0.572 (0.027) | 0.648 (0.041) | 0.500 (0.059) | 0.574 (0.027) |
| **CMU** | 0.669 (0.043) | 0.833 (0.065) | 0.517 (0.056) | 0.675 (0.043) |
| **KKI** | 0.558 (0.027) | 0.329 (0.047) | 0.879 (0.025) | 0.604 (0.024) |
| **LEUVEN** | 0.676 (0.030) | 0.868 (0.040) | 0.452 (0.035) | 0.660 (0.030) |
| **MAX_MUN** | 0.544 (0.024) | 0.588 (0.032) | 0.493 (0.047) | 0.541 (0.025) |
| **NYU** | 0.677 (0.015) | 0.664 (0.027) | 0.693 (0.016) | 0.679 (0.014) |
| **OHSU** | 0.572 (0.040) | 0.643 (0.055) | 0.490 (0.069) | 0.566 (0.041) |
| **OLIN** | 0.658 (0.025) | 0.632 (0.037) | 0.679 (0.040) | 0.655 (0.024) |
| **PITT** | 0.679 (0.022) | 0.816 (0.035) | 0.552 (0.034) | 0.684 (0.023) |
| **SBL** | 0.554 (0.035) | 0.782 (0.039) | 0.325 (0.060) | 0.554 (0.035) |
| **SDSU** | 0.703 (0.023) | 0.768 (0.038) | 0.603 (0.067) | 0.685 (0.028) |
| **STANFORD** | 0.495 (0.034) | 0.214 (0.071) | 0.791 (0.030) | 0.502 (0.033) |
| **TRINITY** | 0.594 (0.029) | 0.584 (0.037) | 0.605 (0.052) | 0.594 (0.030) |
| **UCLA** | 0.700 (0.022) | 0.705 (0.029) | 0.696 (0.024) | 0.700 (0.023) |
| **UM** | 0.692 (0.013) | 0.609 (0.021) | 0.786 (0.021) | 0.697 (0.013) |
| **USM** | 0.725 (0.025) | 0.800 (0.026) | 0.685 (0.036) | 0.742 (0.022) |
| **YALE** | 0.688 (0.024) | 0.682 (0.038) | 0.694 (0.036) | 0.688 (0.024) |
| **Mean** | 0.633 | 0.657 | 0.614 | 0.635 |
| **Weighted Mean** | 0.652 | 0.661 | 0.651 | 0.656 |

# AAL Atlas, Graph features:

Table 2-A. Results using the unchanged correlation matrix. ACC: ACCURACY, SEN: SENSITIVITY, SPE: SPECIFICITY, AUC: Area under curve score, THRESH: Graph density threshold for the reported metrics

| **SITE** | **ACC** | **SEN** | **SPE** | **AUC** |
| --- | --- | --- | --- | --- |
| **CALTECH** | 0.473 (0.036) | 0.538 (0.049) | 0.412 (0.074) | 0.475 (0.036) |
| **CMU** | 0.631 (0.055) | 0.786 (0.086) | 0.487 (0.057) | 0.636 (0.055) |
| **KKI** | 0.488 (0.037) | 0.512 (0.069) | 0.456 (0.094) | 0.484 (0.040) |
| **LEUVEN** | 0.508 (0.019) | 0.761 (0.035) | 0.210 (0.041) | 0.486 (0.019) |
| **MAX_MUN** | 0.537 (0.032) | 0.669 (0.049) | 0.383 (0.050) | 0.526 (0.032) |
| **NYU** | 0.580 (0.018) | 0.547 (0.049) | 0.625 (0.034) | 0.586 (0.015) |
| **OHSU** | 0.426 (0.073) | 0.442 (0.065) | 0.407 (0.118) | 0.425 (0.075) |
| **OLIN** | 0.639 (0.042) | 0.602 (0.069) | 0.669 (0.054) | 0.635 (0.043) |
| **PITT** | 0.513 (0.030) | 0.605 (0.034) | 0.428 (0.061) | 0.516 (0.029) |
| **SBL** | 0.621 (0.055) | 0.566 (0.060) | 0.677 (0.101) | 0.621 (0.055) |
| **SDSU** | 0.620 (0.068) | 0.617 (0.075) | 0.625 (0.073) | 0.621 (0.067) |
| **STANFORD** | 0.506 (0.044) | 0.337 (0.058) | 0.684 (0.077) | 0.511 (0.045) |
| **TRINITY** | 0.503 (0.049) | 0.494 (0.090) | 0.513 (0.056) | 0.503 (0.047) |
| **UCLA** | 0.595 (0.017) | 0.701 (0.034) | 0.509 (0.032) | 0.605 (0.017) |
| **UM** | 0.581 (0.023) | 0.448 (0.060) | 0.73 (0.0380) | 0.589 (0.021) |
| **USM** | 0.565 (0.036) | 0.665 (0.048) | 0.511 (0.048) | 0.588 (0.034) |
| **YALE** | 0.656 (0.032) | 0.622 (0.069) | 0.690 (0.049) | 0.656 (0.032) |
| **Mean** | 0.555 | 0.583 | 0.530 | 0.557 |
| **Weighted Mean** | 0.562 | 0.579 | 0.551 | 0.565 |

Table 2-B. Results using the absolute value of the correlation matrix

| **SITE** | **ACC** | **SEN** | **SPE** | **AUC** |
| --- | --- | --- | --- | --- |
| **CALTECH** | 0.530 (0.028) | 0.525 (0.044) | 0.534 (0.058) | 0.530 (0.028) |
| **CMU** | 0.632 (0.060) | 0.741 (0.066) | 0.531 (0.070) | 0.636 (0.060) |
| **KKI** | 0.541 (0.036) | 0.483 (0.065) | 0.622 (0.071) | 0.553 (0.036) |
| **LEUVEN** | 0.552 (0.030) | 0.708 (0.055) | 0.369 (0.027) | 0.538 (0.028) |
| **MAX_MUN** | 0.532 (0.022) | 0.548 (0.047) | 0.514 (0.040) | 0.531 (0.021) |
| **NYU** | 0.548 (0.016) | 0.534 (0.030) | 0.566 (0.028) | 0.550 (0.015) |
| **OHSU** | 0.537 (0.046) | 0.392 (0.053) | 0.706 (0.064) | 0.549 (0.046) |
| **OLIN** | 0.631 (0.036) | 0.602 (0.092) | 0.653 (0.055) | 0.627 (0.039) |
| **PITT** | 0.522 (0.041) | 0.596 (0.045) | 0.453 (0.057) | 0.524 (0.041) |
| **SBL** | 0.570 (0.056) | 0.627 (0.083) | 0.513 (0.102) | 0.570 (0.056) |
| **SDSU** | 0.615 (0.047) | 0.572 (0.054) | 0.682 (0.066) | 0.627 (0.048) |
| **STANFORD** | 0.611 (0.033) | 0.463 (0.067) | 0.767 (0.068) | 0.615 (0.033) |
| **TRINITY** | 0.591 (0.021) | 0.700 (0.068) | 0.468 (0.059) | 0.584 (0.020) |
| **UCLA** | 0.603 (0.025) | 0.646 (0.045) | 0.568 (0.033) | 0.607 (0.025) |
| **UM** | 0.596 (0.021) | 0.576 (0.034) | 0.617 (0.041) | 0.597 (0.022) |
| **USM** | 0.592 (0.030) | 0.749 (0.045) | 0.507 (0.003) | 0.628 (0.032) |
| **YALE** | 0.642 (0.033) | 0.719 (0.057) | 0.564 (0.060) | 0.642 (0.033) |
| **Mean** | 0.579 | 0.599 | 0.567 | 0.583 |
| **Weighted Mean** | 0.577 | 0.600 | 0.561 | 0.580 |

Table 2-C. Results using the negative correlation matrix

| **SITE** | **ACC** | **SEN** | **SPE** | **AUC** |
| --- | --- | --- | --- | --- |
| **CALTECH** | 0.547 (0.030) | 0.694 (0.061) | 0.409 (0.044) | 0.551 (0.031) |
| **CMU** | 0.689 (0.061) | 0.782 (0.050) | 0.603 (0.109) | 0.692 (0.059) |
| **KKI** | 0.502 (0.051) | 0.464 (0.053) | 0.556 (0.063) | 0.510 (0.0520) |
| **LEUVEN** | 0.568 (0.037) | 0.791 (0.039) | 0.306 (0.053) | 0.548 (0.038) |
| **MAX_MUN** | 0.574 (0.029) | 0.588 (0.049) | 0.558 (0.028) | 0.573 (0.028) |
| **NYU** | 0.588 (0.030) | 0.541 (0.045) | 0.650 (0.049) | 0.595 (0.030) |
| **OHSU** | 0.466 (0.096) | 0.517 (0.075) | 0.406 (0.161) | 0.462 (0.099) |
| **OLIN** | 0.635 (0.044) | 0.618 (0.063) | 0.648 (0.046) | 0.633 (0.045) |
| **PITT** | 0.539 (0.044) | 0.715 (0.046) | 0.375 (0.070) | 0.545 (0.043) |
| **SBL** | 0.619 (0.045) | 0.610 (0.067) | 0.628 (0.066) | 0.619 (0.045) |
| **SDSU** | 0.595 (0.042) | 0.594 (0.055) | 0.596 (0.080) | 0.595 (0.045) |
| **STANFORD** | 0.487 (0.029) | 0.306 (0.045) | 0.678 (0.036) | 0.492 (0.028) |
| **TRINITY** | 0.568 (0.028) | 0.515 (0.067) | 0.628 (0.058) | 0.572 (0.027) |
| **UCLA** | 0.605 (0.03) | 0.730 (0.052) | 0.503 (0.058) | 0.616 (0.029) |
| **UM** | 0.591 (0.020) | 0.477 (0.037) | 0.720 (0.037) | 0.598 (0.020) |
| **USM** | 0.624 (0.038) | 0.741 (0.062) | 0.561 (0.051) | 0.651 (0.039) |
| **YALE** | 0.660 (0.034) | 0.640 (0.051) | 0.679 (0.050) | 0.660 (0.034) |
| **Mean** | 0.580 | 0.607 | 0.559 | 0.583 |
| **Weighted Mean** | 0.584 | 0.600 | 0.578 | 0.589 |

# Craddock 200, Graph features, Only Males:

Table 3-A. Results using the unchanged correlation matrix. ACC: ACCURACY, SEN: SENSITIVITY, SPE: SPECIFICITY, AUC: Area under curve score, THRESH: Graph density threshold for the reported metrics

| **SITE** | **ACC** | **SEN** | **SPE** | **AUC** |
| --- | --- | --- | --- | --- |
| **CALTECH** | 0.519 (0.042) | 0.637 (0.042) | 0.409 (0.069) | 0.523 (0.041) |
| **CMU** | 0.564 (0.046) | 0.878 (0.079) | 0.280 (0.120) | 0.579 (0.044) |
| **KKI** | 0.457 (0.029) | 0.318 (0.084) | 0.632 (0.103) | 0.475 (0.031) |
| **LEUVEN** | 0.567 (0.039) | 0.777 (0.036) | 0.333 (0.053) | 0.555 (0.039) |
| **MAX_MUN** | 0.584 (0.035) | 0.561 (0.053) | 0.614 (0.053) | 0.587 (0.035) |
| **NYU** | 0.616 (0.027) | 0.575 (0.044) | 0.663 (0.032) | 0.619 (0.027) |
| **OHSU** | 0.472 (0.049) | 0.420 (0.134) | 0.533 (0.155) | 0.476 (0.051) |
| **OLIN** | 0.644 (0.042) | 0.493 (0.058) | 0.767 (0.048) | 0.630 (0.042) |
| **PITT** | 0.597 (0.019) | 0.677 (0.031) | 0.523 (0.035) | 0.600 (0.019) |
| **SBL** | 0.610 (0.037) | 0.723 (0.044) | 0.496 (0.051) | 0.610 (0.037) |
| **SDSU** | 0.627 (0.042) | 0.528 (0.052) | 0.748 (0.059) | 0.638 (0.042) |
| **STANFORD** | 0.525 (0.033) | 0.263 (0.027) | 0.805 (0.055) | 0.534 (0.034) |
| **TRINITY** | 0.417 (0.046) | 0.468 (0.043) | 0.360 (0.065) | 0.414 (0.046) |
| **UCLA** | 0.551 (0.036) | 0.596 (0.042) | 0.516 (0.057) | 0.556 (0.035) |
| **UM** | 0.607 (0.022) | 0.492 (0.051) | 0.719 (0.038) | 0.606 (0.023) |
| **USM** | 0.539 (0.046) | 0.665 (0.052) | 0.471 (0.062) | 0.568 (0.043) |
| **YALE** | 0.608 (0.033) | 0.632 (0.053) | 0.583 (0.045) | 0.608 (0.033) |
| **Mean** | 0.559 | 0.571 | 0.556 | 0.563 |
| **Weighted Mean** | 0.570 | 0.570 | 0.578 | 0.574 |

Table 3-B. Results using the absolute value of the correlation matrix

| **SITE** | **ACC** | **SEN** | **SPE** | **AUC** |
| --- | --- | --- | --- | --- |
| **CALTECH** | 0.572 (0.058) | 0.721 (0.062) | 0.432 (0.091) | 0.577 (0.057) |
| **CMU** | 0.502 (0.051) | 0.726 (0.049) | 0.299 (0.100) | 0.513 (0.049) |
| **KKI** | 0.555 (0.045) | 0.439 (0.061) | 0.700 (0.060) | 0.569 (0.045) |
| **LEUVEN** | 0.564 (0.039) | 0.723 (0.049) | 0.387 (0.047) | 0.555 (0.039) |
| **MAX_MUN** | 0.572 (0.028) | 0.522 (0.061) | 0.635 (0.042) | 0.579 (0.025) |
| **NYU** | 0.620 (0.027) | 0.605 (0.036) | 0.638 (0.031) | 0.621 (0.027) |
| **OHSU** | 0.480 (0.041) | 0.413 (0.056) | 0.558 (0.073) | 0.485 (0.042) |
| **OLIN** | 0.557 (0.034) | 0.341 (0.057) | 0.733 (0.042) | 0.537 (0.035) |
| **PITT** | 0.621 (0.034) | 0.604 (0.067) | 0.638 (0.060) | 0.621 (0.035) |
| **SBL** | 0.580 (0.041) | 0.867 (0.048) | 0.292 (0.075) | 0.580 (0.041) |
| **SDSU** | 0.585 (0.036) | 0.540 (0.038) | 0.641 (0.083) | 0.591 (0.039) |
| **STANFORD** | 0.522 (0.029) | 0.250 (0.044) | 0.812 (0.043) | 0.531 (0.029) |
| **TRINITY** | 0.522 (0.030) | 0.551 (0.067) | 0.489 (0.042) | 0.520 (0.028) |
| **UCLA** | 0.601 (0.026) | 0.647 (0.051) | 0.564 (0.038) | 0.606 (0.027) |
| **UM** | 0.628 (0.018) | 0.534 (0.049) | 0.721 (0.039) | 0.627 (0.019) |
| **USM** | 0.601 (0.026) | 0.663 (0.058) | 0.567 (0.041) | 0.615 (0.028) |
| **YALE** | 0.602 (0.029) | 0.699 (0.041) | 0.504 (0.044) | 0.602 (0.029) |
| **Mean** | 0.570 | 0.579 | 0.565 | 0.572 |
| **Weighted Mean** | 0.587 | 0.587 | 0.592 | 0.590 |

Table 3-C. Results using the negative correlation matrix

| **SITE** | **ACC** | **SEN** | **SPE** | **AUC** |
| --- | --- | --- | --- | --- |
| **CALTECH** | 0.560 (0.056) | 0.673 (0.087) | 0.455 (0.081) | 0.564 (0.057) |
| **CMU** | 0.606 (0.066) | 0.791 (0.077) | 0.437 (0.099) | 0.614 (0.065) |
| **KKI** | 0.514 (0.045) | 0.256 (0.056) | 0.836 (0.057) | 0.546 (0.045) |
| **LEUVEN** | 0.592 (0.049) | 0.746 (0.074) | 0.422 (0.049) | 0.584 (0.048) |
| **MAX_MUN** | 0.587 (0.027) | 0.593 (0.038) | 0.581 (0.061) | 0.587 (0.029) |
| **NYU** | 0.590 (0.021) | 0.565 (0.037) | 0.620 (0.026) | 0.592 (0.020) |
| **OHSU** | 0.390 (0.038) | 0.433 (0.052) | 0.340 (0.102) | 0.386 (0.041) |
| **OLIN** | 0.581 (0.049) | 0.504 (0.065) | 0.643 (0.061) | 0.573 (0.050) |
| **PITT** | 0.614 (0.033) | 0.683 (0.045) | 0.550 (0.053) | 0.617 (0.033) |
| **SBL** | 0.603 (0.034) | 0.777 (0.059) | 0.429 (0.048) | 0.603 (0.034) |
| **SDSU** | 0.587 (0.059) | 0.480 (0.077) | 0.719 (0.099) | 0.599 (0.061) |
| **STANFORD** | 0.505 (0.031) | 0.197 (0.047) | 0.833 (0.066) | 0.515 (0.032) |
| **TRINITY** | 0.521 (0.026) | 0.576 (0.047) | 0.459 (0.064) | 0.518 (0.027) |
| **UCLA** | 0.614 (0.025) | 0.607 (0.038) | 0.619 (0.055) | 0.613 (0.023) |
| **UM** | 0.655 (0.026) | 0.565 (0.036) | 0.744 (0.035) | 0.654 (0.026) |
| **USM** | 0.580 (0.026) | 0.758 (0.036) | 0.483 (0.039) | 0.620 (0.024) |
| **YALE** | 0.608 (0.033) | 0.549 (0.067) | 0.667 (0.042) | 0.608 (0.033) |
| **Mean** | 0.571 | 0.574 | 0.579 | 0.576 |
| **Weighted Mean** | 0.587 | 0.580 | 0.604 | 0.592 |

**P-values of statistical test 1 as explained in the main body:**

Table 1-A. p-values between the results from the anticorrelation pipeline and absolute value pipelines. Significant values are shown in green (p<0.05)

|  | **ACC** | **SEN** | **SPE** | **AUC** |
| --- | --- | --- | --- | --- |
| **PITT** | 1 | 0.00704 | 0.049088 | 0.905637 |
| **OLIN** | 0.745423 | 0.090018 | 0.019257 | 0.821816 |
| **OHSU** | 0.264679 | 1 | 0.094141 | 0.214987 |
| **SDSU** | 0.063638 | 0.433303 | 0.00004 | 0.01156 |
| **TRINITY** | 0.555322 | 0.372966 | 0.831455 | 0.573903 |
| **UM** | 0.501903 | 0.869464 | 0.391634 | 0.444736 |
| **USM** | 0.024404 | 0.033199 | 0.29734 | 0.006976 |
| **YALE** | 0.026911 | 0.001852 | 0.000223 | 0.026911 |
| **CMU** | 0.193015 | 0.300643 | 1 | 0.192664 |
| **LEUVEN** | 0.00028 | 0.242119 | 0.013745 | 0.000275 |
| **KKI** | 0.420509 | 0.849664 | 0.143648 | 0.304904 |
| **NYU** | 0.099042 | 0.149582 | 0.627434 | 0.130409 |
| **STANFORD** | 1 | 0.017178 | 0.041921 | 0.854016 |
| **UCLA** | 0.077851 | 0.070563 | 0.23401 | 0.070927 |
| **MAX_MUN** | 0.545763 | 0.03776 | 0.03403 | 0.390427 |
| **CALTECH** | 1 | 0.077971 | 0.375713 | 0.962579 |
| **SBL** | 0.012924 | 0.242876 | 0.00619 | 0.012924 |

Table 2-B. p-values between the results from the anticorrelation pipeline and positive pipelines. Significant values are shown in green (p<0.05)

|  | **ACC** | **SEN** | **SPE** | **AUC** |
| --- | --- | --- | --- | --- |
| **PITT** | 0.111491 | 0.001875 | 0.758752 | 0.095194 |
| **OLIN** | 0.266627 | 1 | 0.195389 | 0.34826 |
| **OHSU** | 0.014456 | 0.000045 | 1 | 0.024348 |
| **SDSU** | 0.321425 | 0.137486 | 0.060976 | 0.062476 |
| **TRINITY** | 0.065748 | 0.054074 | 0.175743 | 0.069965 |
| **UM** | 0.309386 | 0.389027 | 0.038197 | 0.243787 |
| **USM** | 0.0319 | 0.0203 | 0.097081 | 0.014103 |
| **YALE** | 0.481453 | 0.038982 | 0.020157 | 0.481453 |
| **CMU** | 0.360802 | 0.308386 | 0.103791 | 0.42871 |
| **LEUVEN** | 0.127988 | 0.074681 | 0.698401 | 0.144683 |
| **KKI** | 0.019064 | 0.028193 | 0.332427 | 0.023005 |
| **NYU** | 0.014188 | 0.015801 | 0.447723 | 0.020558 |
| **STANFORD** | 0.143854 | 0.083119 | 1 | 0.151991 |
| **UCLA** | 0.002746 | 0.423281 | 0.001345 | 0.003386 |
| **MAX_MUN** | 0.521641 | 0.37543 | 0.060718 | 0.403319 |
| **CALTECH** | 0.369812 | 0.412668 | 0.193263 | 0.388424 |
| **SBL** | 0.466957 | 0.206151 | 1 | 0.466957 |

# Performance of other models:

Table 1 POS pipeline, Only graph features

|  |  | **accuracy** | **sensitivity** | **specificity** | **auc** |
| --- | --- | --- | --- | --- | --- |
| **SDSU** | LogisticRegression | 0.59 | 0.54 | 0.67 | 0.61 |
| **SDSU** | RFClassifier | 0.63 | 0.55 | 0.75 | 0.65 |
| **SDSU** | SVM-RBF | 0.66 | 0.60 | 0.75 | 0.67 |
| **TRINITY** | LogisticRegression | 0.41 | 0.42 | 0.40 | 0.41 |
| **TRINITY** | RFClassifier | 0.48 | 0.50 | 0.46 | 0.48 |
| **TRINITY** | SVM-RBF | 0.43 | 0.52 | 0.33 | 0.42 |
| **UM** | LogisticRegression | 0.58 | 0.53 | 0.63 | 0.58 |
| **UM** | RFClassifier | 0.62 | 0.52 | 0.72 | 0.62 |
| **UM** | SVM-RBF | 0.64 | 0.52 | 0.78 | 0.65 |
| **USM** | LogisticRegression | 0.53 | 0.64 | 0.47 | 0.56 |
| **USM** | RFClassifier | 0.53 | 0.68 | 0.45 | 0.56 |
| **USM** | SVM-RBF | 0.55 | 0.74 | 0.45 | 0.59 |
| **YALE** | LogisticRegression | 0.58 | 0.59 | 0.56 | 0.58 |
| **YALE** | RFClassifier | 0.58 | 0.61 | 0.54 | 0.58 |
| **YALE** | SVM-RBF | 0.63 | 0.66 | 0.60 | 0.63 |
| **CMU** | LogisticRegression | 0.49 | 0.55 | 0.42 | 0.49 |
| **CMU** | RFClassifier | 0.58 | 0.76 | 0.41 | 0.59 |
| **CMU** | SVM-RBF | 0.67 | 0.69 | 0.65 | 0.67 |
| **LEUVEN** | LogisticRegression | 0.61 | 0.78 | 0.41 | 0.60 |
| **LEUVEN** | RFClassifier | 0.57 | 0.80 | 0.31 | 0.55 |
| **LEUVEN** | SVM-RBF | 0.53 | 0.76 | 0.27 | 0.51 |
| **KKI** | LogisticRegression | 0.55 | 0.47 | 0.66 | 0.57 |
| **KKI** | RFClassifier | 0.52 | 0.37 | 0.73 | 0.55 |
| **KKI** | SVM-RBF | 0.55 | 0.37 | 0.81 | 0.59 |
| **NYU** | LogisticRegression | 0.60 | 0.62 | 0.56 | 0.59 |
| **NYU** | RFClassifier | 0.63 | 0.63 | 0.61 | 0.62 |
| **NYU** | SVM-RBF | 0.64 | 0.59 | 0.71 | 0.65 |
| **STANFORD** | LogisticRegression | 0.52 | 0.44 | 0.61 | 0.52 |
| **STANFORD** | RFClassifier | 0.50 | 0.25 | 0.77 | 0.51 |
| **STANFORD** | SVM-RBF | 0.56 | 0.29 | 0.84 | 0.56 |
| **UCLA** | LogisticRegression | 0.59 | 0.62 | 0.57 | 0.59 |
| **UCLA** | RFClassifier | 0.61 | 0.69 | 0.55 | 0.62 |
| **UCLA** | SVM-RBF | 0.62 | 0.71 | 0.54 | 0.63 |
| **MAX_MUN** | LogisticRegression | 0.57 | 0.51 | 0.65 | 0.58 |
| **MAX_MUN** | RFClassifier | 0.53 | 0.62 | 0.42 | 0.52 |
| **MAX_MUN** | SVM-RBF | 0.53 | 0.60 | 0.46 | 0.53 |
| **CALTECH** | LogisticRegression | 0.60 | 0.75 | 0.46 | 0.60 |
| **CALTECH** | RFClassifier | 0.49 | 0.59 | 0.39 | 0.49 |
| **CALTECH** | SVM-RBF | 0.50 | 0.60 | 0.40 | 0.50 |
| **SBL** | LogisticRegression | 0.54 | 0.57 | 0.52 | 0.54 |
| **SBL** | RFClassifier | 0.61 | 0.76 | 0.47 | 0.61 |
| **SBL** | SVM-RBF | 0.61 | 0.77 | 0.45 | 0.61 |
| **OLIN** | LogisticRegression | 0.56 | 0.40 | 0.68 | 0.54 |
| **OLIN** | RFClassifier | 0.56 | 0.57 | 0.54 | 0.56 |
| **OLIN** | SVM-RBF | 0.63 | 0.59 | 0.66 | 0.62 |
| **OHSU** | LogisticRegression | 0.56 | 0.55 | 0.57 | 0.56 |
| **OHSU** | RFClassifier | 0.49 | 0.59 | 0.37 | 0.48 |
| **OHSU** | SVM-RBF | 0.46 | 0.51 | 0.39 | 0.45 |
| **PITT** | LogisticRegression | 0.49 | 0.62 | 0.38 | 0.50 |
| **PITT** | RFClassifier | 0.56 | 0.66 | 0.46 | 0.56 |
| **PITT** | SVM-RBF | 0.55 | 0.64 | 0.47 | 0.55 |

Table 2 POS pipeline, graph + PCA features

|  |  | **accuracy** | **sensitivity** | **specificity** | **auc** |
| --- | --- | --- | --- | --- | --- |
| **SDSU** | LogisticRegression | 0.69 | 0.74 | 0.61 | 0.67 |
| **SDSU** | RFClassifier | 0.65 | 0.64 | 0.66 | 0.65 |
| **SDSU** | SVM-RBF | 0.68 | 0.72 | 0.61 | 0.67 |
| **TRINITY** | LogisticRegression | 0.41 | 0.40 | 0.43 | 0.41 |
| **TRINITY** | RFClassifier | 0.49 | 0.56 | 0.41 | 0.49 |
| **TRINITY** | SVM-RBF | 0.45 | 0.60 | 0.27 | 0.44 |
| **UM** | LogisticRegression | 0.63 | 0.58 | 0.68 | 0.63 |
| **UM** | RFClassifier | 0.62 | 0.52 | 0.73 | 0.63 |
| **UM** | SVM-RBF | 0.68 | 0.64 | 0.71 | 0.68 |
| **USM** | LogisticRegression | 0.67 | 0.74 | 0.64 | 0.69 |
| **USM** | RFClassifier | 0.52 | 0.79 | 0.37 | 0.58 |
| **USM** | SVM-RBF | 0.55 | 0.82 | 0.41 | 0.61 |
| **YALE** | LogisticRegression | 0.73 | 0.74 | 0.72 | 0.73 |
| **YALE** | RFClassifier | 0.60 | 0.71 | 0.49 | 0.60 |
| **YALE** | SVM-RBF | 0.66 | 0.75 | 0.56 | 0.66 |
| **CMU** | LogisticRegression | 0.63 | 0.84 | 0.43 | 0.63 |
| **CMU** | RFClassifier | 0.56 | 0.85 | 0.30 | 0.57 |
| **CMU** | SVM-RBF | 0.66 | 0.69 | 0.64 | 0.67 |
| **LEUVEN** | LogisticRegression | 0.62 | 0.83 | 0.37 | 0.60 |
| **LEUVEN** | RFClassifier | 0.59 | 0.86 | 0.28 | 0.57 |
| **LEUVEN** | SVM-RBF | 0.57 | 0.86 | 0.23 | 0.55 |
| **KKI** | LogisticRegression | 0.51 | 0.38 | 0.69 | 0.53 |
| **KKI** | RFClassifier | 0.52 | 0.41 | 0.68 | 0.54 |
| **KKI** | SVM-RBF | 0.58 | 0.42 | 0.80 | 0.61 |
| **NYU** | LogisticRegression | 0.70 | 0.68 | 0.72 | 0.70 |
| **NYU** | RFClassifier | 0.63 | 0.68 | 0.56 | 0.62 |
| **NYU** | SVM-RBF | 0.67 | 0.73 | 0.60 | 0.66 |
| **STANFORD** | LogisticRegression | 0.54 | 0.32 | 0.77 | 0.54 |
| **STANFORD** | RFClassifier | 0.53 | 0.30 | 0.77 | 0.54 |
| **STANFORD** | SVM-RBF | 0.59 | 0.36 | 0.83 | 0.59 |
| **UCLA** | LogisticRegression | 0.64 | 0.63 | 0.65 | 0.64 |
| **UCLA** | RFClassifier | 0.60 | 0.76 | 0.46 | 0.61 |
| **UCLA** | SVM-RBF | 0.64 | 0.81 | 0.50 | 0.66 |
| **MAX_MUN** | LogisticRegression | 0.54 | 0.56 | 0.53 | 0.54 |
| **MAX_MUN** | RFClassifier | 0.51 | 0.67 | 0.32 | 0.49 |
| **MAX_MUN** | SVM-RBF | 0.52 | 0.65 | 0.36 | 0.51 |
| **CALTECH** | LogisticRegression | 0.64 | 0.68 | 0.61 | 0.64 |
| **CALTECH** | RFClassifier | 0.56 | 0.70 | 0.41 | 0.56 |
| **CALTECH** | SVM-RBF | 0.52 | 0.68 | 0.37 | 0.52 |
| **SBL** | LogisticRegression | 0.52 | 0.73 | 0.31 | 0.52 |
| **SBL** | RFClassifier | 0.58 | 0.83 | 0.33 | 0.58 |
| **SBL** | SVM-RBF | 0.55 | 0.90 | 0.20 | 0.55 |
| **OLIN** | LogisticRegression | 0.66 | 0.53 | 0.76 | 0.65 |
| **OLIN** | RFClassifier | 0.55 | 0.62 | 0.49 | 0.56 |
| **OLIN** | SVM-RBF | 0.59 | 0.62 | 0.57 | 0.59 |
| **OHSU** | LogisticRegression | 0.64 | 0.71 | 0.56 | 0.64 |
| **OHSU** | RFClassifier | 0.48 | 0.59 | 0.36 | 0.47 |
| **OHSU** | SVM-RBF | 0.43 | 0.66 | 0.16 | 0.41 |
| **PITT** | LogisticRegression | 0.61 | 0.76 | 0.46 | 0.61 |
| **PITT** | RFClassifier | 0.55 | 0.68 | 0.42 | 0.55 |
| **PITT** | SVM-RBF | 0.58 | 0.74 | 0.44 | 0.59 |

Table 3 NEG pipeline, only graph features

|  |  | **accuracy** | **sensitivity** | **specificity** | **auc** |
| --- | --- | --- | --- | --- | --- |
| **SDSU** | LogisticRegression | 0.62 | 0.65 | 0.57 | 0.61 |
| **SDSU** | RFClassifier | 0.61 | 0.57 | 0.67 | 0.62 |
| **SDSU** | SVM-RBF | 0.65 | 0.57 | 0.76 | 0.67 |
| **TRINITY** | LogisticRegression | 0.48 | 0.52 | 0.44 | 0.48 |
| **TRINITY** | RFClassifier | 0.58 | 0.59 | 0.56 | 0.57 |
| **TRINITY** | SVM-RBF | 0.51 | 0.60 | 0.41 | 0.50 |
| **UM** | LogisticRegression | 0.62 | 0.55 | 0.70 | 0.62 |
| **UM** | RFClassifier | 0.62 | 0.52 | 0.73 | 0.63 |
| **UM** | SVM-RBF | 0.66 | 0.57 | 0.77 | 0.67 |
| **USM** | LogisticRegression | 0.58 | 0.70 | 0.51 | 0.61 |
| **USM** | RFClassifier | 0.60 | 0.66 | 0.57 | 0.62 |
| **USM** | SVM-RBF | 0.60 | 0.83 | 0.48 | 0.65 |
| **YALE** | LogisticRegression | 0.56 | 0.53 | 0.60 | 0.56 |
| **YALE** | RFClassifier | 0.59 | 0.56 | 0.63 | 0.59 |
| **YALE** | SVM-RBF | 0.62 | 0.57 | 0.66 | 0.62 |
| **CMU** | LogisticRegression | 0.50 | 0.55 | 0.46 | 0.51 |
| **CMU** | RFClassifier | 0.66 | 0.81 | 0.51 | 0.66 |
| **CMU** | SVM-RBF | 0.62 | 0.73 | 0.51 | 0.62 |
| **LEUVEN** | LogisticRegression | 0.55 | 0.63 | 0.47 | 0.55 |
| **LEUVEN** | RFClassifier | 0.59 | 0.77 | 0.38 | 0.58 |
| **LEUVEN** | SVM-RBF | 0.58 | 0.79 | 0.33 | 0.56 |
| **KKI** | LogisticRegression | 0.56 | 0.46 | 0.69 | 0.58 |
| **KKI** | RFClassifier | 0.46 | 0.24 | 0.77 | 0.51 |
| **KKI** | SVM-RBF | 0.52 | 0.29 | 0.85 | 0.57 |
| **NYU** | LogisticRegression | 0.59 | 0.58 | 0.60 | 0.59 |
| **NYU** | RFClassifier | 0.61 | 0.60 | 0.62 | 0.61 |
| **NYU** | SVM-RBF | 0.62 | 0.58 | 0.68 | 0.63 |
| **STANFORD** | LogisticRegression | 0.60 | 0.52 | 0.68 | 0.60 |
| **STANFORD** | RFClassifier | 0.48 | 0.22 | 0.76 | 0.49 |
| **STANFORD** | SVM-RBF | 0.49 | 0.21 | 0.79 | 0.50 |
| **UCLA** | LogisticRegression | 0.57 | 0.58 | 0.56 | 0.57 |
| **UCLA** | RFClassifier | 0.62 | 0.69 | 0.56 | 0.63 |
| **UCLA** | SVM-RBF | 0.64 | 0.74 | 0.56 | 0.65 |
| **MAX_MUN** | LogisticRegression | 0.56 | 0.53 | 0.59 | 0.56 |
| **MAX_MUN** | RFClassifier | 0.53 | 0.59 | 0.46 | 0.53 |
| **MAX_MUN** | SVM-RBF | 0.53 | 0.59 | 0.46 | 0.52 |
| **CALTECH** | LogisticRegression | 0.55 | 0.58 | 0.52 | 0.55 |
| **CALTECH** | RFClassifier | 0.52 | 0.72 | 0.33 | 0.53 |
| **CALTECH** | SVM-RBF | 0.49 | 0.64 | 0.36 | 0.50 |
| **SBL** | LogisticRegression | 0.56 | 0.71 | 0.42 | 0.56 |
| **SBL** | RFClassifier | 0.63 | 0.80 | 0.46 | 0.63 |
| **SBL** | SVM-RBF | 0.61 | 0.80 | 0.41 | 0.61 |
| **OLIN** | LogisticRegression | 0.57 | 0.59 | 0.55 | 0.57 |
| **OLIN** | RFClassifier | 0.58 | 0.58 | 0.58 | 0.58 |
| **OLIN** | SVM-RBF | 0.62 | 0.64 | 0.60 | 0.62 |
| **OHSU** | LogisticRegression | 0.47 | 0.52 | 0.42 | 0.47 |
| **OHSU** | RFClassifier | 0.40 | 0.52 | 0.26 | 0.39 |
| **OHSU** | SVM-RBF | 0.39 | 0.47 | 0.29 | 0.38 |
| **PITT** | LogisticRegression | 0.55 | 0.67 | 0.44 | 0.56 |
| **PITT** | RFClassifier | 0.56 | 0.72 | 0.41 | 0.57 |
| **PITT** | SVM-RBF | 0.57 | 0.72 | 0.44 | 0.58 |

Table 4 NEG pipeline, graph + PCA features

|  |  | **accuracy** | **sensitivity** | **specificity** | **auc** |
| --- | --- | --- | --- | --- | --- |
| **SDSU** | LogisticRegression | 0.71 | 0.77 | 0.61 | 0.69 |
| **SDSU** | RFClassifier | 0.63 | 0.65 | 0.59 | 0.62 |
| **SDSU** | SVM-RBF | 0.68 | 0.72 | 0.61 | 0.67 |
| **TRINITY** | LogisticRegression | 0.56 | 0.54 | 0.58 | 0.56 |
| **TRINITY** | RFClassifier | 0.58 | 0.63 | 0.52 | 0.57 |
| **TRINITY** | SVM-RBF | 0.49 | 0.66 | 0.30 | 0.48 |
| **UM** | LogisticRegression | 0.67 | 0.60 | 0.75 | 0.68 |
| **UM** | RFClassifier | 0.66 | 0.56 | 0.77 | 0.66 |
| **UM** | SVM-RBF | 0.69 | 0.66 | 0.72 | 0.69 |
| **USM** | LogisticRegression | 0.67 | 0.73 | 0.64 | 0.68 |
| **USM** | RFClassifier | 0.59 | 0.81 | 0.48 | 0.64 |
| **USM** | SVM-RBF | 0.59 | 0.88 | 0.43 | 0.65 |
| **YALE** | LogisticRegression | 0.69 | 0.67 | 0.71 | 0.69 |
| **YALE** | RFClassifier | 0.61 | 0.63 | 0.58 | 0.61 |
| **YALE** | SVM-RBF | 0.64 | 0.65 | 0.63 | 0.64 |
| **CMU** | LogisticRegression | 0.61 | 0.81 | 0.43 | 0.62 |
| **CMU** | RFClassifier | 0.59 | 0.84 | 0.36 | 0.60 |
| **CMU** | SVM-RBF | 0.64 | 0.75 | 0.54 | 0.64 |
| **LEUVEN** | LogisticRegression | 0.65 | 0.80 | 0.47 | 0.64 |
| **LEUVEN** | RFClassifier | 0.60 | 0.83 | 0.33 | 0.58 |
| **LEUVEN** | SVM-RBF | 0.61 | 0.87 | 0.31 | 0.59 |
| **KKI** | LogisticRegression | 0.55 | 0.32 | 0.87 | 0.59 |
| **KKI** | RFClassifier | 0.50 | 0.27 | 0.82 | 0.54 |
| **KKI** | SVM-RBF | 0.57 | 0.37 | 0.85 | 0.61 |
| **NYU** | LogisticRegression | 0.66 | 0.65 | 0.68 | 0.66 |
| **NYU** | RFClassifier | 0.62 | 0.65 | 0.58 | 0.62 |
| **NYU** | SVM-RBF | 0.68 | 0.71 | 0.64 | 0.67 |
| **STANFORD** | LogisticRegression | 0.63 | 0.43 | 0.84 | 0.64 |
| **STANFORD** | RFClassifier | 0.42 | 0.15 | 0.71 | 0.43 |
| **STANFORD** | SVM-RBF | 0.52 | 0.29 | 0.76 | 0.53 |
| **UCLA** | LogisticRegression | 0.67 | 0.65 | 0.69 | 0.67 |
| **UCLA** | RFClassifier | 0.62 | 0.75 | 0.52 | 0.63 |
| **UCLA** | SVM-RBF | 0.65 | 0.82 | 0.52 | 0.67 |
| **MAX_MUN** | LogisticRegression | 0.53 | 0.54 | 0.53 | 0.53 |
| **MAX_MUN** | RFClassifier | 0.50 | 0.62 | 0.36 | 0.49 |
| **MAX_MUN** | SVM-RBF | 0.48 | 0.62 | 0.31 | 0.47 |
| **CALTECH** | LogisticRegression | 0.64 | 0.67 | 0.62 | 0.64 |
| **CALTECH** | RFClassifier | 0.56 | 0.81 | 0.32 | 0.57 |
| **CALTECH** | SVM-RBF | 0.49 | 0.66 | 0.33 | 0.50 |
| **SBL** | LogisticRegression | 0.53 | 0.73 | 0.32 | 0.53 |
| **SBL** | RFClassifier | 0.56 | 0.79 | 0.33 | 0.56 |
| **SBL** | SVM-RBF | 0.57 | 0.85 | 0.28 | 0.57 |
| **OLIN** | LogisticRegression | 0.60 | 0.54 | 0.65 | 0.59 |
| **OLIN** | RFClassifier | 0.54 | 0.60 | 0.49 | 0.55 |
| **OLIN** | SVM-RBF | 0.61 | 0.70 | 0.54 | 0.62 |
| **OHSU** | LogisticRegression | 0.59 | 0.70 | 0.46 | 0.58 |
| **OHSU** | RFClassifier | 0.39 | 0.54 | 0.21 | 0.38 |
| **OHSU** | SVM-RBF | 0.42 | 0.67 | 0.13 | 0.40 |
| **PITT** | LogisticRegression | 0.68 | 0.83 | 0.55 | 0.69 |
| **PITT** | RFClassifier | 0.55 | 0.81 | 0.31 | 0.56 |
| **PITT** | SVM-RBF | 0.59 | 0.83 | 0.36 | 0.60 |

Table 5 ABS pipeline, only graph features

|  |  | **accuracy** | **sensitivity** | **specificity** | **auc** |
| --- | --- | --- | --- | --- | --- |
| **SDSU** | LogisticRegression | 0.54 | 0.57 | 0.50 | 0.53 |
| **SDSU** | RFClassifier | 0.63 | 0.61 | 0.66 | 0.63 |
| **SDSU** | SVM-RBF | 0.62 | 0.65 | 0.58 | 0.61 |
| **TRINITY** | LogisticRegression | 0.50 | 0.46 | 0.55 | 0.50 |
| **TRINITY** | RFClassifier | 0.53 | 0.56 | 0.49 | 0.52 |
| **TRINITY** | SVM-RBF | 0.52 | 0.59 | 0.44 | 0.51 |
| **UM** | LogisticRegression | 0.59 | 0.55 | 0.64 | 0.60 |
| **UM** | RFClassifier | 0.62 | 0.51 | 0.75 | 0.63 |
| **UM** | SVM-RBF | 0.68 | 0.58 | 0.79 | 0.68 |
| **USM** | LogisticRegression | 0.53 | 0.57 | 0.51 | 0.54 |
| **USM** | RFClassifier | 0.61 | 0.80 | 0.51 | 0.65 |
| **USM** | SVM-RBF | 0.66 | 0.76 | 0.60 | 0.68 |
| **YALE** | LogisticRegression | 0.60 | 0.77 | 0.42 | 0.60 |
| **YALE** | RFClassifier | 0.60 | 0.73 | 0.47 | 0.60 |
| **YALE** | SVM-RBF | 0.61 | 0.77 | 0.46 | 0.61 |
| **CMU** | LogisticRegression | 0.52 | 0.68 | 0.38 | 0.53 |
| **CMU** | RFClassifier | 0.61 | 0.82 | 0.42 | 0.62 |
| **CMU** | SVM-RBF | 0.60 | 0.85 | 0.37 | 0.61 |
| **LEUVEN** | LogisticRegression | 0.50 | 0.59 | 0.39 | 0.49 |
| **LEUVEN** | RFClassifier | 0.56 | 0.81 | 0.27 | 0.54 |
| **LEUVEN** | SVM-RBF | 0.55 | 0.78 | 0.28 | 0.53 |
| **KKI** | LogisticRegression | 0.54 | 0.56 | 0.51 | 0.54 |
| **KKI** | RFClassifier | 0.48 | 0.36 | 0.65 | 0.50 |
| **KKI** | SVM-RBF | 0.51 | 0.42 | 0.64 | 0.53 |
| **NYU** | LogisticRegression | 0.60 | 0.58 | 0.62 | 0.60 |
| **NYU** | RFClassifier | 0.61 | 0.61 | 0.60 | 0.60 |
| **NYU** | SVM-RBF | 0.65 | 0.65 | 0.65 | 0.65 |
| **STANFORD** | LogisticRegression | 0.57 | 0.49 | 0.64 | 0.57 |
| **STANFORD** | RFClassifier | 0.52 | 0.26 | 0.78 | 0.52 |
| **STANFORD** | SVM-RBF | 0.54 | 0.27 | 0.83 | 0.55 |
| **UCLA** | LogisticRegression | 0.58 | 0.59 | 0.58 | 0.58 |
| **UCLA** | RFClassifier | 0.59 | 0.68 | 0.51 | 0.60 |
| **UCLA** | SVM-RBF | 0.64 | 0.67 | 0.62 | 0.64 |
| **MAX_MUN** | LogisticRegression | 0.48 | 0.49 | 0.47 | 0.48 |
| **MAX_MUN** | RFClassifier | 0.49 | 0.56 | 0.40 | 0.48 |
| **MAX_MUN** | SVM-RBF | 0.56 | 0.57 | 0.54 | 0.56 |
| **CALTECH** | LogisticRegression | 0.53 | 0.53 | 0.52 | 0.53 |
| **CALTECH** | RFClassifier | 0.51 | 0.61 | 0.41 | 0.51 |
| **CALTECH** | SVM-RBF | 0.44 | 0.66 | 0.24 | 0.45 |
| **SBL** | LogisticRegression | 0.49 | 0.65 | 0.33 | 0.49 |
| **SBL** | RFClassifier | 0.52 | 0.73 | 0.30 | 0.52 |
| **SBL** | SVM-RBF | 0.57 | 0.90 | 0.23 | 0.57 |
| **OLIN** | LogisticRegression | 0.58 | 0.55 | 0.60 | 0.58 |
| **OLIN** | RFClassifier | 0.65 | 0.57 | 0.70 | 0.64 |
| **OLIN** | SVM-RBF | 0.64 | 0.50 | 0.74 | 0.62 |
| **OHSU** | LogisticRegression | 0.54 | 0.54 | 0.53 | 0.54 |
| **OHSU** | RFClassifier | 0.47 | 0.43 | 0.51 | 0.47 |
| **OHSU** | SVM-RBF | 0.46 | 0.44 | 0.47 | 0.46 |
| **PITT** | LogisticRegression | 0.53 | 0.53 | 0.54 | 0.53 |
| **PITT** | RFClassifier | 0.55 | 0.58 | 0.52 | 0.55 |
| **PITT** | SVM-RBF | 0.57 | 0.56 | 0.58 | 0.57 |

Table 6 ABS pipeline, graph + PCA features

|  |  | **accuracy** | **sensitivity** | **specificity** | **auc** |
| --- | --- | --- | --- | --- | --- |
| **SDSU** | LogisticRegression | 0.62 | 0.66 | 0.56 | 0.61 |
| **SDSU** | RFClassifier | 0.63 | 0.69 | 0.52 | 0.61 |
| **SDSU** | SVM-RBF | 0.63 | 0.70 | 0.53 | 0.61 |
| **TRINITY** | LogisticRegression | 0.52 | 0.49 | 0.55 | 0.52 |
| **TRINITY** | RFClassifier | 0.54 | 0.61 | 0.46 | 0.54 |
| **TRINITY** | SVM-RBF | 0.55 | 0.65 | 0.44 | 0.54 |
| **UM** | LogisticRegression | 0.68 | 0.62 | 0.75 | 0.69 |
| **UM** | RFClassifier | 0.62 | 0.54 | 0.71 | 0.63 |
| **UM** | SVM-RBF | 0.70 | 0.61 | 0.79 | 0.70 |
| **USM** | LogisticRegression | 0.67 | 0.68 | 0.66 | 0.67 |
| **USM** | RFClassifier | 0.57 | 0.85 | 0.42 | 0.63 |
| **USM** | SVM-RBF | 0.66 | 0.82 | 0.57 | 0.69 |
| **YALE** | LogisticRegression | 0.68 | 0.79 | 0.58 | 0.68 |
| **YALE** | RFClassifier | 0.59 | 0.75 | 0.43 | 0.59 |
| **YALE** | SVM-RBF | 0.66 | 0.82 | 0.50 | 0.66 |
| **CMU** | LogisticRegression | 0.65 | 0.88 | 0.43 | 0.65 |
| **CMU** | RFClassifier | 0.58 | 0.88 | 0.30 | 0.59 |
| **CMU** | SVM-RBF | 0.63 | 0.74 | 0.54 | 0.64 |
| **LEUVEN** | LogisticRegression | 0.61 | 0.79 | 0.40 | 0.59 |
| **LEUVEN** | RFClassifier | 0.58 | 0.87 | 0.24 | 0.55 |
| **LEUVEN** | SVM-RBF | 0.58 | 0.86 | 0.26 | 0.56 |
| **KKI** | LogisticRegression | 0.55 | 0.46 | 0.67 | 0.56 |
| **KKI** | RFClassifier | 0.52 | 0.43 | 0.64 | 0.54 |
| **KKI** | SVM-RBF | 0.54 | 0.44 | 0.68 | 0.56 |
| **NYU** | LogisticRegression | 0.67 | 0.66 | 0.68 | 0.67 |
| **NYU** | RFClassifier | 0.64 | 0.71 | 0.54 | 0.62 |
| **NYU** | SVM-RBF | 0.68 | 0.73 | 0.61 | 0.67 |
| **STANFORD** | LogisticRegression | 0.59 | 0.39 | 0.80 | 0.59 |
| **STANFORD** | RFClassifier | 0.54 | 0.31 | 0.77 | 0.54 |
| **STANFORD** | SVM-RBF | 0.53 | 0.26 | 0.81 | 0.53 |
| **UCLA** | LogisticRegression | 0.66 | 0.66 | 0.66 | 0.66 |
| **UCLA** | RFClassifier | 0.60 | 0.77 | 0.47 | 0.62 |
| **UCLA** | SVM-RBF | 0.65 | 0.75 | 0.57 | 0.66 |
| **MAX_MUN** | LogisticRegression | 0.49 | 0.52 | 0.47 | 0.49 |
| **MAX_MUN** | RFClassifier | 0.51 | 0.69 | 0.30 | 0.49 |
| **MAX_MUN** | SVM-RBF | 0.54 | 0.58 | 0.49 | 0.54 |
| **CALTECH** | LogisticRegression | 0.61 | 0.65 | 0.58 | 0.61 |
| **CALTECH** | RFClassifier | 0.51 | 0.70 | 0.32 | 0.51 |
| **CALTECH** | SVM-RBF | 0.51 | 0.63 | 0.40 | 0.52 |
| **SBL** | LogisticRegression | 0.52 | 0.80 | 0.25 | 0.52 |
| **SBL** | RFClassifier | 0.56 | 0.87 | 0.24 | 0.56 |
| **SBL** | SVM-RBF | 0.55 | 0.93 | 0.16 | 0.55 |
| **OLIN** | LogisticRegression | 0.65 | 0.59 | 0.69 | 0.64 |
| **OLIN** | RFClassifier | 0.65 | 0.70 | 0.62 | 0.66 |
| **OLIN** | SVM-RBF | 0.71 | 0.62 | 0.78 | 0.70 |
| **OHSU** | LogisticRegression | 0.63 | 0.65 | 0.61 | 0.63 |
| **OHSU** | RFClassifier | 0.46 | 0.50 | 0.41 | 0.46 |
| **OHSU** | SVM-RBF | 0.42 | 0.49 | 0.33 | 0.41 |
| **PITT** | LogisticRegression | 0.65 | 0.69 | 0.61 | 0.65 |
| **PITT** | RFClassifier | 0.55 | 0.65 | 0.46 | 0.55 |
| **PITT** | SVM-RBF | 0.63 | 0.71 | 0.55 | 0.63 |
