# Supplementary material for: The Importance of Anti-correlations in Graph Theory Based Classification of Autism Spectrum Disorder
Source: Front Neurosci. 2020 Aug 7;14:676. doi: 10.3389/fnins.2020.00676 (PMC7426475; doi:10.3389/fnins.2020.00676)
Supplement: Supplementary file 3 [file Table_3.DOCX]

Supplementary Material

# Craddock 200 Atlas, PCA + Graph features:

**Table 1. Results with graph features + PCA feature**. ACC: ACCURACY; SEN: SENSITIVITY; SPE: SPECIFICITY; AUC: Area under curve score; Pos: Unchanged pipeline; Neg: Anti-correlation pipeline; Abs: Absolute value pipeline

# AAL Atlas, Graph features:

**Table 2. Results with only graph features based on the AAL atlas**

# Craddock 200, Graph features, Only Males:

**Table 3. Results with only graph features based on only male subjects**

# P-values of statistical test 1 as explained in the main body:

Table 4.1. p-values between the results from the anticorrelation pipeline and absolute value pipelines. Significant values are shown in green (p<0.05). Corrected for multiple comparisons using the Holm-Sidak method

|  | **ACC** | **SEN** | **SPE** | **AUC** |
| --- | --- | --- | --- | --- |
| **PITT** | 1.000 | 0.014 | 0.140 | 0.906 |
| **OLIN** | 0.745 | 0.172 | 0.057 | 0.822 |
| **OHSU** | 0.265 | 1.000 | 0.257 | 0.356 |
| **SDSU** | 0.179 | 0.679 | 0.000 | 0.034 |
| **TRINITY** | 0.555 | 0.373 | 0.831 | 0.574 |
| **UM** | 0.752 | 0.896 | 0.630 | 0.692 |
| **USM** | 0.071 | 0.065 | 0.506 | 0.021 |
| **YALE** | 0.079 | 0.006 | 0.001 | 0.079 |
| **CMU** | 0.474 | 0.511 | 1.000 | 0.474 |
| **LEUVEN** | 0.001 | 0.426 | 0.041 | 0.001 |
| **KKI** | 0.421 | 0.850 | 0.372 | 0.305 |
| **NYU** | 0.188 | 0.270 | 0.861 | 0.244 |
| **STANFORD** | 1.000 | 0.034 | 0.082 | 0.854 |
| **UCLA** | 0.078 | 0.197 | 0.234 | 0.136 |
| **MAX_MUN** | 0.891 | 0.109 | 0.099 | 0.773 |
| **CALTECH** | 1.000 | 0.150 | 0.376 | 0.963 |
| **SBL** | 0.038 | 0.500 | 0.018 | 0.038 |

Table 4.2. p-values between the results from the anticorrelation pipeline and positive pipelines. Significant values are shown in green (p<0.05). Corrected for multiple comparisons using the Holm-Sidak method

|  | **ACC** | **SEN** | **SPE** | **AUC** |
| --- | --- | --- | --- | --- |
| **PITT** | 0.211 | 0.006 | 0.759 | 0.181 |
| **OLIN** | 0.500 | 1.000 | 0.353 | 0.575 |
| **OHSU** | 0.043 | 0.000 | 1.000 | 0.071 |
| **SDSU** | 0.321 | 0.358 | 0.118 | 0.121 |
| **TRINITY** | 0.157 | 0.154 | 0.439 | 0.172 |
| **UM** | 0.671 | 0.772 | 0.110 | 0.568 |
| **USM** | 0.071 | 0.060 | 0.264 | 0.028 |
| **YALE** | 0.481 | 0.039 | 0.020 | 0.481 |
| **CMU** | 0.591 | 0.511 | 0.197 | 0.649 |
| **LEUVEN** | 0.128 | 0.208 | 0.698 | 0.145 |
| **KKI** | 0.056 | 0.082 | 0.554 | 0.067 |
| **NYU** | 0.042 | 0.047 | 0.832 | 0.060 |
| **STANFORD** | 0.346 | 0.083 | 1.000 | 0.390 |
| **UCLA** | 0.008 | 0.667 | 0.003 | 0.010 |
| **MAX_MUN** | 0.891 | 0.565 | 0.118 | 0.773 |
| **CALTECH** | 0.750 | 0.413 | 0.349 | 0.771 |
| **SBL** | 0.467 | 0.500 | 1.000 | 0.467 |

# Performance of other models:

Table 5.1 POS pipeline, Only graph features

|  |  | **accuracy** | **sensitivity** | **specificity** | **auc** |
| --- | --- | --- | --- | --- | --- |
| **SDSU** | LogisticRegression | 0.59 | 0.54 | 0.67 | 0.61 |
| **SDSU** | RFClassifier | 0.63 | 0.55 | 0.75 | 0.65 |
| **SDSU** | SVM-RBF | 0.66 | 0.60 | 0.75 | 0.67 |
| **TRINITY** | LogisticRegression | 0.41 | 0.42 | 0.40 | 0.41 |
| **TRINITY** | RFClassifier | 0.48 | 0.50 | 0.46 | 0.48 |
| **TRINITY** | SVM-RBF | 0.43 | 0.52 | 0.33 | 0.42 |
| **UM** | LogisticRegression | 0.58 | 0.53 | 0.63 | 0.58 |
| **UM** | RFClassifier | 0.62 | 0.52 | 0.72 | 0.62 |
| **UM** | SVM-RBF | 0.64 | 0.52 | 0.78 | 0.65 |
| **USM** | LogisticRegression | 0.53 | 0.64 | 0.47 | 0.56 |
| **USM** | RFClassifier | 0.53 | 0.68 | 0.45 | 0.56 |
| **USM** | SVM-RBF | 0.55 | 0.74 | 0.45 | 0.59 |
| **YALE** | LogisticRegression | 0.58 | 0.59 | 0.56 | 0.58 |
| **YALE** | RFClassifier | 0.58 | 0.61 | 0.54 | 0.58 |
| **YALE** | SVM-RBF | 0.63 | 0.66 | 0.60 | 0.63 |
| **CMU** | LogisticRegression | 0.49 | 0.55 | 0.42 | 0.49 |
| **CMU** | RFClassifier | 0.58 | 0.76 | 0.41 | 0.59 |
| **CMU** | SVM-RBF | 0.67 | 0.69 | 0.65 | 0.67 |
| **LEUVEN** | LogisticRegression | 0.61 | 0.78 | 0.41 | 0.60 |
| **LEUVEN** | RFClassifier | 0.57 | 0.80 | 0.31 | 0.55 |
| **LEUVEN** | SVM-RBF | 0.53 | 0.76 | 0.27 | 0.51 |
| **KKI** | LogisticRegression | 0.55 | 0.47 | 0.66 | 0.57 |
| **KKI** | RFClassifier | 0.52 | 0.37 | 0.73 | 0.55 |
| **KKI** | SVM-RBF | 0.55 | 0.37 | 0.81 | 0.59 |
| **NYU** | LogisticRegression | 0.60 | 0.62 | 0.56 | 0.59 |
| **NYU** | RFClassifier | 0.63 | 0.63 | 0.61 | 0.62 |
| **NYU** | SVM-RBF | 0.64 | 0.59 | 0.71 | 0.65 |
| **STANFORD** | LogisticRegression | 0.52 | 0.44 | 0.61 | 0.52 |
| **STANFORD** | RFClassifier | 0.50 | 0.25 | 0.77 | 0.51 |
| **STANFORD** | SVM-RBF | 0.56 | 0.29 | 0.84 | 0.56 |
| **UCLA** | LogisticRegression | 0.59 | 0.62 | 0.57 | 0.59 |
| **UCLA** | RFClassifier | 0.61 | 0.69 | 0.55 | 0.62 |
| **UCLA** | SVM-RBF | 0.62 | 0.71 | 0.54 | 0.63 |
| **MAX_MUN** | LogisticRegression | 0.57 | 0.51 | 0.65 | 0.58 |
| **MAX_MUN** | RFClassifier | 0.53 | 0.62 | 0.42 | 0.52 |
| **MAX_MUN** | SVM-RBF | 0.53 | 0.60 | 0.46 | 0.53 |
| **CALTECH** | LogisticRegression | 0.60 | 0.75 | 0.46 | 0.60 |
| **CALTECH** | RFClassifier | 0.49 | 0.59 | 0.39 | 0.49 |
| **CALTECH** | SVM-RBF | 0.50 | 0.60 | 0.40 | 0.50 |
| **SBL** | LogisticRegression | 0.54 | 0.57 | 0.52 | 0.54 |
| **SBL** | RFClassifier | 0.61 | 0.76 | 0.47 | 0.61 |
| **SBL** | SVM-RBF | 0.61 | 0.77 | 0.45 | 0.61 |
| **OLIN** | LogisticRegression | 0.56 | 0.40 | 0.68 | 0.54 |
| **OLIN** | RFClassifier | 0.56 | 0.57 | 0.54 | 0.56 |
| **OLIN** | SVM-RBF | 0.63 | 0.59 | 0.66 | 0.62 |
| **OHSU** | LogisticRegression | 0.56 | 0.55 | 0.57 | 0.56 |
| **OHSU** | RFClassifier | 0.49 | 0.59 | 0.37 | 0.48 |
| **OHSU** | SVM-RBF | 0.46 | 0.51 | 0.39 | 0.45 |
| **PITT** | LogisticRegression | 0.49 | 0.62 | 0.38 | 0.50 |
| **PITT** | RFClassifier | 0.56 | 0.66 | 0.46 | 0.56 |
| **PITT** | SVM-RBF | 0.55 | 0.64 | 0.47 | 0.55 |

Table 5.2 POS pipeline, graph + PCA features

|  |  | **accuracy** | **sensitivity** | **specificity** | **auc** |
| --- | --- | --- | --- | --- | --- |
| **SDSU** | LogisticRegression | 0.69 | 0.74 | 0.61 | 0.67 |
| **SDSU** | RFClassifier | 0.65 | 0.64 | 0.66 | 0.65 |
| **SDSU** | SVM-RBF | 0.68 | 0.72 | 0.61 | 0.67 |
| **TRINITY** | LogisticRegression | 0.41 | 0.40 | 0.43 | 0.41 |
| **TRINITY** | RFClassifier | 0.49 | 0.56 | 0.41 | 0.49 |
| **TRINITY** | SVM-RBF | 0.45 | 0.60 | 0.27 | 0.44 |
| **UM** | LogisticRegression | 0.63 | 0.58 | 0.68 | 0.63 |
| **UM** | RFClassifier | 0.62 | 0.52 | 0.73 | 0.63 |
| **UM** | SVM-RBF | 0.68 | 0.64 | 0.71 | 0.68 |
| **USM** | LogisticRegression | 0.67 | 0.74 | 0.64 | 0.69 |
| **USM** | RFClassifier | 0.52 | 0.79 | 0.37 | 0.58 |
| **USM** | SVM-RBF | 0.55 | 0.82 | 0.41 | 0.61 |
| **YALE** | LogisticRegression | 0.73 | 0.74 | 0.72 | 0.73 |
| **YALE** | RFClassifier | 0.60 | 0.71 | 0.49 | 0.60 |
| **YALE** | SVM-RBF | 0.66 | 0.75 | 0.56 | 0.66 |
| **CMU** | LogisticRegression | 0.63 | 0.84 | 0.43 | 0.63 |
| **CMU** | RFClassifier | 0.56 | 0.85 | 0.30 | 0.57 |
| **CMU** | SVM-RBF | 0.66 | 0.69 | 0.64 | 0.67 |
| **LEUVEN** | LogisticRegression | 0.62 | 0.83 | 0.37 | 0.60 |
| **LEUVEN** | RFClassifier | 0.59 | 0.86 | 0.28 | 0.57 |
| **LEUVEN** | SVM-RBF | 0.57 | 0.86 | 0.23 | 0.55 |
| **KKI** | LogisticRegression | 0.51 | 0.38 | 0.69 | 0.53 |
| **KKI** | RFClassifier | 0.52 | 0.41 | 0.68 | 0.54 |
| **KKI** | SVM-RBF | 0.58 | 0.42 | 0.80 | 0.61 |
| **NYU** | LogisticRegression | 0.70 | 0.68 | 0.72 | 0.70 |
| **NYU** | RFClassifier | 0.63 | 0.68 | 0.56 | 0.62 |
| **NYU** | SVM-RBF | 0.67 | 0.73 | 0.60 | 0.66 |
| **STANFORD** | LogisticRegression | 0.54 | 0.32 | 0.77 | 0.54 |
| **STANFORD** | RFClassifier | 0.53 | 0.30 | 0.77 | 0.54 |
| **STANFORD** | SVM-RBF | 0.59 | 0.36 | 0.83 | 0.59 |
| **UCLA** | LogisticRegression | 0.64 | 0.63 | 0.65 | 0.64 |
| **UCLA** | RFClassifier | 0.60 | 0.76 | 0.46 | 0.61 |
| **UCLA** | SVM-RBF | 0.64 | 0.81 | 0.50 | 0.66 |
| **MAX_MUN** | LogisticRegression | 0.54 | 0.56 | 0.53 | 0.54 |
| **MAX_MUN** | RFClassifier | 0.51 | 0.67 | 0.32 | 0.49 |
| **MAX_MUN** | SVM-RBF | 0.52 | 0.65 | 0.36 | 0.51 |
| **CALTECH** | LogisticRegression | 0.64 | 0.68 | 0.61 | 0.64 |
| **CALTECH** | RFClassifier | 0.56 | 0.70 | 0.41 | 0.56 |
| **CALTECH** | SVM-RBF | 0.52 | 0.68 | 0.37 | 0.52 |
| **SBL** | LogisticRegression | 0.52 | 0.73 | 0.31 | 0.52 |
| **SBL** | RFClassifier | 0.58 | 0.83 | 0.33 | 0.58 |
| **SBL** | SVM-RBF | 0.55 | 0.90 | 0.20 | 0.55 |
| **OLIN** | LogisticRegression | 0.66 | 0.53 | 0.76 | 0.65 |
| **OLIN** | RFClassifier | 0.55 | 0.62 | 0.49 | 0.56 |
| **OLIN** | SVM-RBF | 0.59 | 0.62 | 0.57 | 0.59 |
| **OHSU** | LogisticRegression | 0.64 | 0.71 | 0.56 | 0.64 |
| **OHSU** | RFClassifier | 0.48 | 0.59 | 0.36 | 0.47 |
| **OHSU** | SVM-RBF | 0.43 | 0.66 | 0.16 | 0.41 |
| **PITT** | LogisticRegression | 0.61 | 0.76 | 0.46 | 0.61 |
| **PITT** | RFClassifier | 0.55 | 0.68 | 0.42 | 0.55 |
| **PITT** | SVM-RBF | 0.58 | 0.74 | 0.44 | 0.59 |

Table 5.3 NEG pipeline, only graph features

|  |  | **accuracy** | **sensitivity** | **specificity** | **auc** |
| --- | --- | --- | --- | --- | --- |
| **SDSU** | LogisticRegression | 0.62 | 0.65 | 0.57 | 0.61 |
| **SDSU** | RFClassifier | 0.61 | 0.57 | 0.67 | 0.62 |
| **SDSU** | SVM-RBF | 0.65 | 0.57 | 0.76 | 0.67 |
| **TRINITY** | LogisticRegression | 0.48 | 0.52 | 0.44 | 0.48 |
| **TRINITY** | RFClassifier | 0.58 | 0.59 | 0.56 | 0.57 |
| **TRINITY** | SVM-RBF | 0.51 | 0.60 | 0.41 | 0.50 |
| **UM** | LogisticRegression | 0.62 | 0.55 | 0.70 | 0.62 |
| **UM** | RFClassifier | 0.62 | 0.52 | 0.73 | 0.63 |
| **UM** | SVM-RBF | 0.66 | 0.57 | 0.77 | 0.67 |
| **USM** | LogisticRegression | 0.58 | 0.70 | 0.51 | 0.61 |
| **USM** | RFClassifier | 0.60 | 0.66 | 0.57 | 0.62 |
| **USM** | SVM-RBF | 0.60 | 0.83 | 0.48 | 0.65 |
| **YALE** | LogisticRegression | 0.56 | 0.53 | 0.60 | 0.56 |
| **YALE** | RFClassifier | 0.59 | 0.56 | 0.63 | 0.59 |
| **YALE** | SVM-RBF | 0.62 | 0.57 | 0.66 | 0.62 |
| **CMU** | LogisticRegression | 0.50 | 0.55 | 0.46 | 0.51 |
| **CMU** | RFClassifier | 0.66 | 0.81 | 0.51 | 0.66 |
| **CMU** | SVM-RBF | 0.62 | 0.73 | 0.51 | 0.62 |
| **LEUVEN** | LogisticRegression | 0.55 | 0.63 | 0.47 | 0.55 |
| **LEUVEN** | RFClassifier | 0.59 | 0.77 | 0.38 | 0.58 |
| **LEUVEN** | SVM-RBF | 0.58 | 0.79 | 0.33 | 0.56 |
| **KKI** | LogisticRegression | 0.56 | 0.46 | 0.69 | 0.58 |
| **KKI** | RFClassifier | 0.46 | 0.24 | 0.77 | 0.51 |
| **KKI** | SVM-RBF | 0.52 | 0.29 | 0.85 | 0.57 |
| **NYU** | LogisticRegression | 0.59 | 0.58 | 0.60 | 0.59 |
| **NYU** | RFClassifier | 0.61 | 0.60 | 0.62 | 0.61 |
| **NYU** | SVM-RBF | 0.62 | 0.58 | 0.68 | 0.63 |
| **STANFORD** | LogisticRegression | 0.60 | 0.52 | 0.68 | 0.60 |
| **STANFORD** | RFClassifier | 0.48 | 0.22 | 0.76 | 0.49 |
| **STANFORD** | SVM-RBF | 0.49 | 0.21 | 0.79 | 0.50 |
| **UCLA** | LogisticRegression | 0.57 | 0.58 | 0.56 | 0.57 |
| **UCLA** | RFClassifier | 0.62 | 0.69 | 0.56 | 0.63 |
| **UCLA** | SVM-RBF | 0.64 | 0.74 | 0.56 | 0.65 |
| **MAX_MUN** | LogisticRegression | 0.56 | 0.53 | 0.59 | 0.56 |
| **MAX_MUN** | RFClassifier | 0.53 | 0.59 | 0.46 | 0.53 |
| **MAX_MUN** | SVM-RBF | 0.53 | 0.59 | 0.46 | 0.52 |
| **CALTECH** | LogisticRegression | 0.55 | 0.58 | 0.52 | 0.55 |
| **CALTECH** | RFClassifier | 0.52 | 0.72 | 0.33 | 0.53 |
| **CALTECH** | SVM-RBF | 0.49 | 0.64 | 0.36 | 0.50 |
| **SBL** | LogisticRegression | 0.56 | 0.71 | 0.42 | 0.56 |
| **SBL** | RFClassifier | 0.63 | 0.80 | 0.46 | 0.63 |
| **SBL** | SVM-RBF | 0.61 | 0.80 | 0.41 | 0.61 |
| **OLIN** | LogisticRegression | 0.57 | 0.59 | 0.55 | 0.57 |
| **OLIN** | RFClassifier | 0.58 | 0.58 | 0.58 | 0.58 |
| **OLIN** | SVM-RBF | 0.62 | 0.64 | 0.60 | 0.62 |
| **OHSU** | LogisticRegression | 0.47 | 0.52 | 0.42 | 0.47 |
| **OHSU** | RFClassifier | 0.40 | 0.52 | 0.26 | 0.39 |
| **OHSU** | SVM-RBF | 0.39 | 0.47 | 0.29 | 0.38 |
| **PITT** | LogisticRegression | 0.55 | 0.67 | 0.44 | 0.56 |
| **PITT** | RFClassifier | 0.56 | 0.72 | 0.41 | 0.57 |
| **PITT** | SVM-RBF | 0.57 | 0.72 | 0.44 | 0.58 |

Table 5.4 NEG pipeline, graph + PCA features

|  |  | **accuracy** | **sensitivity** | **specificity** | **auc** |
| --- | --- | --- | --- | --- | --- |
| **SDSU** | LogisticRegression | 0.71 | 0.77 | 0.61 | 0.69 |
| **SDSU** | RFClassifier | 0.63 | 0.65 | 0.59 | 0.62 |
| **SDSU** | SVM-RBF | 0.68 | 0.72 | 0.61 | 0.67 |
| **TRINITY** | LogisticRegression | 0.56 | 0.54 | 0.58 | 0.56 |
| **TRINITY** | RFClassifier | 0.58 | 0.63 | 0.52 | 0.57 |
| **TRINITY** | SVM-RBF | 0.49 | 0.66 | 0.30 | 0.48 |
| **UM** | LogisticRegression | 0.67 | 0.60 | 0.75 | 0.68 |
| **UM** | RFClassifier | 0.66 | 0.56 | 0.77 | 0.66 |
| **UM** | SVM-RBF | 0.69 | 0.66 | 0.72 | 0.69 |
| **USM** | LogisticRegression | 0.67 | 0.73 | 0.64 | 0.68 |
| **USM** | RFClassifier | 0.59 | 0.81 | 0.48 | 0.64 |
| **USM** | SVM-RBF | 0.59 | 0.88 | 0.43 | 0.65 |
| **YALE** | LogisticRegression | 0.69 | 0.67 | 0.71 | 0.69 |
| **YALE** | RFClassifier | 0.61 | 0.63 | 0.58 | 0.61 |
| **YALE** | SVM-RBF | 0.64 | 0.65 | 0.63 | 0.64 |
| **CMU** | LogisticRegression | 0.61 | 0.81 | 0.43 | 0.62 |
| **CMU** | RFClassifier | 0.59 | 0.84 | 0.36 | 0.60 |
| **CMU** | SVM-RBF | 0.64 | 0.75 | 0.54 | 0.64 |
| **LEUVEN** | LogisticRegression | 0.65 | 0.80 | 0.47 | 0.64 |
| **LEUVEN** | RFClassifier | 0.60 | 0.83 | 0.33 | 0.58 |
| **LEUVEN** | SVM-RBF | 0.61 | 0.87 | 0.31 | 0.59 |
| **KKI** | LogisticRegression | 0.55 | 0.32 | 0.87 | 0.59 |
| **KKI** | RFClassifier | 0.50 | 0.27 | 0.82 | 0.54 |
| **KKI** | SVM-RBF | 0.57 | 0.37 | 0.85 | 0.61 |
| **NYU** | LogisticRegression | 0.66 | 0.65 | 0.68 | 0.66 |
| **NYU** | RFClassifier | 0.62 | 0.65 | 0.58 | 0.62 |
| **NYU** | SVM-RBF | 0.68 | 0.71 | 0.64 | 0.67 |
| **STANFORD** | LogisticRegression | 0.63 | 0.43 | 0.84 | 0.64 |
| **STANFORD** | RFClassifier | 0.42 | 0.15 | 0.71 | 0.43 |
| **STANFORD** | SVM-RBF | 0.52 | 0.29 | 0.76 | 0.53 |
| **UCLA** | LogisticRegression | 0.67 | 0.65 | 0.69 | 0.67 |
| **UCLA** | RFClassifier | 0.62 | 0.75 | 0.52 | 0.63 |
| **UCLA** | SVM-RBF | 0.65 | 0.82 | 0.52 | 0.67 |
| **MAX_MUN** | LogisticRegression | 0.53 | 0.54 | 0.53 | 0.53 |
| **MAX_MUN** | RFClassifier | 0.50 | 0.62 | 0.36 | 0.49 |
| **MAX_MUN** | SVM-RBF | 0.48 | 0.62 | 0.31 | 0.47 |
| **CALTECH** | LogisticRegression | 0.64 | 0.67 | 0.62 | 0.64 |
| **CALTECH** | RFClassifier | 0.56 | 0.81 | 0.32 | 0.57 |
| **CALTECH** | SVM-RBF | 0.49 | 0.66 | 0.33 | 0.50 |
| **SBL** | LogisticRegression | 0.53 | 0.73 | 0.32 | 0.53 |
| **SBL** | RFClassifier | 0.56 | 0.79 | 0.33 | 0.56 |
| **SBL** | SVM-RBF | 0.57 | 0.85 | 0.28 | 0.57 |
| **OLIN** | LogisticRegression | 0.60 | 0.54 | 0.65 | 0.59 |
| **OLIN** | RFClassifier | 0.54 | 0.60 | 0.49 | 0.55 |
| **OLIN** | SVM-RBF | 0.61 | 0.70 | 0.54 | 0.62 |
| **OHSU** | LogisticRegression | 0.59 | 0.70 | 0.46 | 0.58 |
| **OHSU** | RFClassifier | 0.39 | 0.54 | 0.21 | 0.38 |
| **OHSU** | SVM-RBF | 0.42 | 0.67 | 0.13 | 0.40 |
| **PITT** | LogisticRegression | 0.68 | 0.83 | 0.55 | 0.69 |
| **PITT** | RFClassifier | 0.55 | 0.81 | 0.31 | 0.56 |
| **PITT** | SVM-RBF | 0.59 | 0.83 | 0.36 | 0.60 |

Table 5.5 ABS pipeline, only graph features

|  |  | **accuracy** | **sensitivity** | **specificity** | **auc** |
| --- | --- | --- | --- | --- | --- |
| **SDSU** | LogisticRegression | 0.54 | 0.57 | 0.50 | 0.53 |
| **SDSU** | RFClassifier | 0.63 | 0.61 | 0.66 | 0.63 |
| **SDSU** | SVM-RBF | 0.62 | 0.65 | 0.58 | 0.61 |
| **TRINITY** | LogisticRegression | 0.50 | 0.46 | 0.55 | 0.50 |
| **TRINITY** | RFClassifier | 0.53 | 0.56 | 0.49 | 0.52 |
| **TRINITY** | SVM-RBF | 0.52 | 0.59 | 0.44 | 0.51 |
| **UM** | LogisticRegression | 0.59 | 0.55 | 0.64 | 0.60 |
| **UM** | RFClassifier | 0.62 | 0.51 | 0.75 | 0.63 |
| **UM** | SVM-RBF | 0.68 | 0.58 | 0.79 | 0.68 |
| **USM** | LogisticRegression | 0.53 | 0.57 | 0.51 | 0.54 |
| **USM** | RFClassifier | 0.61 | 0.80 | 0.51 | 0.65 |
| **USM** | SVM-RBF | 0.66 | 0.76 | 0.60 | 0.68 |
| **YALE** | LogisticRegression | 0.60 | 0.77 | 0.42 | 0.60 |
| **YALE** | RFClassifier | 0.60 | 0.73 | 0.47 | 0.60 |
| **YALE** | SVM-RBF | 0.61 | 0.77 | 0.46 | 0.61 |
| **CMU** | LogisticRegression | 0.52 | 0.68 | 0.38 | 0.53 |
| **CMU** | RFClassifier | 0.61 | 0.82 | 0.42 | 0.62 |
| **CMU** | SVM-RBF | 0.60 | 0.85 | 0.37 | 0.61 |
| **LEUVEN** | LogisticRegression | 0.50 | 0.59 | 0.39 | 0.49 |
| **LEUVEN** | RFClassifier | 0.56 | 0.81 | 0.27 | 0.54 |
| **LEUVEN** | SVM-RBF | 0.55 | 0.78 | 0.28 | 0.53 |
| **KKI** | LogisticRegression | 0.54 | 0.56 | 0.51 | 0.54 |
| **KKI** | RFClassifier | 0.48 | 0.36 | 0.65 | 0.50 |
| **KKI** | SVM-RBF | 0.51 | 0.42 | 0.64 | 0.53 |
| **NYU** | LogisticRegression | 0.60 | 0.58 | 0.62 | 0.60 |
| **NYU** | RFClassifier | 0.61 | 0.61 | 0.60 | 0.60 |
| **NYU** | SVM-RBF | 0.65 | 0.65 | 0.65 | 0.65 |
| **STANFORD** | LogisticRegression | 0.57 | 0.49 | 0.64 | 0.57 |
| **STANFORD** | RFClassifier | 0.52 | 0.26 | 0.78 | 0.52 |
| **STANFORD** | SVM-RBF | 0.54 | 0.27 | 0.83 | 0.55 |
| **UCLA** | LogisticRegression | 0.58 | 0.59 | 0.58 | 0.58 |
| **UCLA** | RFClassifier | 0.59 | 0.68 | 0.51 | 0.60 |
| **UCLA** | SVM-RBF | 0.64 | 0.67 | 0.62 | 0.64 |
| **MAX_MUN** | LogisticRegression | 0.48 | 0.49 | 0.47 | 0.48 |
| **MAX_MUN** | RFClassifier | 0.49 | 0.56 | 0.40 | 0.48 |
| **MAX_MUN** | SVM-RBF | 0.56 | 0.57 | 0.54 | 0.56 |
| **CALTECH** | LogisticRegression | 0.53 | 0.53 | 0.52 | 0.53 |
| **CALTECH** | RFClassifier | 0.51 | 0.61 | 0.41 | 0.51 |
| **CALTECH** | SVM-RBF | 0.44 | 0.66 | 0.24 | 0.45 |
| **SBL** | LogisticRegression | 0.49 | 0.65 | 0.33 | 0.49 |
| **SBL** | RFClassifier | 0.52 | 0.73 | 0.30 | 0.52 |
| **SBL** | SVM-RBF | 0.57 | 0.90 | 0.23 | 0.57 |
| **OLIN** | LogisticRegression | 0.58 | 0.55 | 0.60 | 0.58 |
| **OLIN** | RFClassifier | 0.65 | 0.57 | 0.70 | 0.64 |
| **OLIN** | SVM-RBF | 0.64 | 0.50 | 0.74 | 0.62 |
| **OHSU** | LogisticRegression | 0.54 | 0.54 | 0.53 | 0.54 |
| **OHSU** | RFClassifier | 0.47 | 0.43 | 0.51 | 0.47 |
| **OHSU** | SVM-RBF | 0.46 | 0.44 | 0.47 | 0.46 |
| **PITT** | LogisticRegression | 0.53 | 0.53 | 0.54 | 0.53 |
| **PITT** | RFClassifier | 0.55 | 0.58 | 0.52 | 0.55 |
| **PITT** | SVM-RBF | 0.57 | 0.56 | 0.58 | 0.57 |

Table 5.6 ABS pipeline, graph + PCA features

|  |  | **accuracy** | **sensitivity** | **specificity** | **auc** |
| --- | --- | --- | --- | --- | --- |
| **SDSU** | LogisticRegression | 0.62 | 0.66 | 0.56 | 0.61 |
| **SDSU** | RFClassifier | 0.63 | 0.69 | 0.52 | 0.61 |
| **SDSU** | SVM-RBF | 0.63 | 0.70 | 0.53 | 0.61 |
| **TRINITY** | LogisticRegression | 0.52 | 0.49 | 0.55 | 0.52 |
| **TRINITY** | RFClassifier | 0.54 | 0.61 | 0.46 | 0.54 |
| **TRINITY** | SVM-RBF | 0.55 | 0.65 | 0.44 | 0.54 |
| **UM** | LogisticRegression | 0.68 | 0.62 | 0.75 | 0.69 |
| **UM** | RFClassifier | 0.62 | 0.54 | 0.71 | 0.63 |
| **UM** | SVM-RBF | 0.70 | 0.61 | 0.79 | 0.70 |
| **USM** | LogisticRegression | 0.67 | 0.68 | 0.66 | 0.67 |
| **USM** | RFClassifier | 0.57 | 0.85 | 0.42 | 0.63 |
| **USM** | SVM-RBF | 0.66 | 0.82 | 0.57 | 0.69 |
| **YALE** | LogisticRegression | 0.68 | 0.79 | 0.58 | 0.68 |
| **YALE** | RFClassifier | 0.59 | 0.75 | 0.43 | 0.59 |
| **YALE** | SVM-RBF | 0.66 | 0.82 | 0.50 | 0.66 |
| **CMU** | LogisticRegression | 0.65 | 0.88 | 0.43 | 0.65 |
| **CMU** | RFClassifier | 0.58 | 0.88 | 0.30 | 0.59 |
| **CMU** | SVM-RBF | 0.63 | 0.74 | 0.54 | 0.64 |
| **LEUVEN** | LogisticRegression | 0.61 | 0.79 | 0.40 | 0.59 |
| **LEUVEN** | RFClassifier | 0.58 | 0.87 | 0.24 | 0.55 |
| **LEUVEN** | SVM-RBF | 0.58 | 0.86 | 0.26 | 0.56 |
| **KKI** | LogisticRegression | 0.55 | 0.46 | 0.67 | 0.56 |
| **KKI** | RFClassifier | 0.52 | 0.43 | 0.64 | 0.54 |
| **KKI** | SVM-RBF | 0.54 | 0.44 | 0.68 | 0.56 |
| **NYU** | LogisticRegression | 0.67 | 0.66 | 0.68 | 0.67 |
| **NYU** | RFClassifier | 0.64 | 0.71 | 0.54 | 0.62 |
| **NYU** | SVM-RBF | 0.68 | 0.73 | 0.61 | 0.67 |
| **STANFORD** | LogisticRegression | 0.59 | 0.39 | 0.80 | 0.59 |
| **STANFORD** | RFClassifier | 0.54 | 0.31 | 0.77 | 0.54 |
| **STANFORD** | SVM-RBF | 0.53 | 0.26 | 0.81 | 0.53 |
| **UCLA** | LogisticRegression | 0.66 | 0.66 | 0.66 | 0.66 |
| **UCLA** | RFClassifier | 0.60 | 0.77 | 0.47 | 0.62 |
| **UCLA** | SVM-RBF | 0.65 | 0.75 | 0.57 | 0.66 |
| **MAX_MUN** | LogisticRegression | 0.49 | 0.52 | 0.47 | 0.49 |
| **MAX_MUN** | RFClassifier | 0.51 | 0.69 | 0.30 | 0.49 |
| **MAX_MUN** | SVM-RBF | 0.54 | 0.58 | 0.49 | 0.54 |
| **CALTECH** | LogisticRegression | 0.61 | 0.65 | 0.58 | 0.61 |
| **CALTECH** | RFClassifier | 0.51 | 0.70 | 0.32 | 0.51 |
| **CALTECH** | SVM-RBF | 0.51 | 0.63 | 0.40 | 0.52 |
| **SBL** | LogisticRegression | 0.52 | 0.80 | 0.25 | 0.52 |
| **SBL** | RFClassifier | 0.56 | 0.87 | 0.24 | 0.56 |
| **SBL** | SVM-RBF | 0.55 | 0.93 | 0.16 | 0.55 |
| **OLIN** | LogisticRegression | 0.65 | 0.59 | 0.69 | 0.64 |
| **OLIN** | RFClassifier | 0.65 | 0.70 | 0.62 | 0.66 |
| **OLIN** | SVM-RBF | 0.71 | 0.62 | 0.78 | 0.70 |
| **OHSU** | LogisticRegression | 0.63 | 0.65 | 0.61 | 0.63 |
| **OHSU** | RFClassifier | 0.46 | 0.50 | 0.41 | 0.46 |
| **OHSU** | SVM-RBF | 0.42 | 0.49 | 0.33 | 0.41 |
| **PITT** | LogisticRegression | 0.65 | 0.69 | 0.61 | 0.65 |
| **PITT** | RFClassifier | 0.55 | 0.65 | 0.46 | 0.55 |
| **PITT** | SVM-RBF | 0.63 | 0.71 | 0.55 | 0.63 |

# Graph Metrics

The measures of integration used in the study are Global/Local Characteristic Path Length $\left( L/L_{i} \right)$ and Global/Local Efficiency $\left( E/E_{loc} \right)$. If we define the shortest path between two nodes as the least number of edges connecting those two nodes, $\left( L \right)$ would be the average of all the shortest paths. $\left( L_{i} \right)$ would be the average shortest path of node $i$ to all other nodes in the network. Likewise, if we average the inverse of the shortest path we would get $\left( E \right)$. $\left( E_{loc,i} \right)$ is calculated by calculating the efficiency on the subnetwork defined by the neighborhood of node $i$.

The measures of segregation in this study are Global/Local Clustering Coefficient $\left( {C/C}_{i} \right)$. The local metric is calculated as the ratio of a nodes neighbors that are neighbors of eachother to all the neighboring nodes. Averaging this over all nodes derives the global metric. Thus, nodes with low nodal degree will have the same contribution to the overall clustering coefficient as higher degree nodes. Transitivity $\left( T \right)$ tries to normalize the contributions based on the nodal degree of the nodes, so higher degree nodes would be more prevalent in determining the modularity of the network. Another measure, Assortativity $(-1<A<1)$, is positive when, on average, similar degree nodes tend to connect to each other and is negative when higher degree nodes tend to connect more with lower degree nodes.

Finally, several centrality measures were calculated. Betweenness Centrality$\left( BC\left( v \right) \right)$ is local metric counting the number of shortest paths of the network that go through a node $v$. Within Module Z-score $z_{i}$ quantifies the centrality of each node within its own subnetwork or module. Thus, a node with high within module Z-score is an important node for within module information flow. Participation Coefficient $y_{i}$ quantifies the centrality of a node in a module to that of the nodes in another module. A high participation coefficient signifies importance in between module communications. Finally, Eigenvector $x_{v}$ centrality is another measure of a nodes “hubness” and calculates the centrality of a node based on the centrality of its neighbors.

Overall the breakdown of the number of features per metric per atlas is as follows:

Table 6.1. Number of features per metric per atlas

| **Metric** | **# Features AAL** | **# Features CC-200** |
| --- | --- | --- |
| **Betweenness Centrality** | 116 | 200 |
| **Within Module Z-score** | 116 | 200 |
| **Participation Coefficient** | 116 | 200 |
| **Eigenvector centrality** | 116 | 200 |
| **Local Characteristic Path Length** | 116 | 200 |
| **Local Efficiency** | 116 | 200 |
| **Local Clustering Coefficient** | 116 | 200 |
| **Transitivity** | 1 | 1 |
| **Assortativity** | 1 | 1 |
| **Global Characteristic Path Length** | 1 | 1 |
| **Global Clustering Coefficient** | 1 | 1 |
| **Sum** | 816 | 1404 |
